# Characterization of 3D Printing on Jute Fabrics

**DOI:** 10.3390/polym13193202

**Published:** 2021-09-22

**Authors:** Edgar Adrián Franco-Urquiza, Yael Ramírez Escamilla, Perla Itzel Alcántara Llanas

**Affiliations:** 1 National Council for Science and Technology (CONACYT—CIDESI), Center for Engineering and Industrial Development, Carretera Estatal 200, Querétaro 76265, Mexico; 2 Department of Mechatronics, Center for Engineering and Industrial Development (CIDESI), Carretera Estatal 200, Querétaro 76265, Mexico; c.yael.r.escamilla@gmail.com (Y.R.E.); perla.alcantara@cidesi.edu.mx (P.I.A.L.)

**Keywords:** jute fabrics, 3D printing, mechanical properties, eco-friendly composites

## Abstract

This work evaluates the feasibility to manufacture polylactic acid (PLA) composites using jute fiber fabrics. For characterization, PLA-fused filament was successfully deposed onto jute fabrics to print dog-bone tensile specimens (Type I specimen from ASTM D638). The jute fabrics were chemically modified, treated with flame retardant additives, and sprayed with aerosol adhesive to improve the mechanical properties of PLA/Jute fabric composites. The elastic modulus and the strength of PLA were higher than PLA composites, and the plastic deformation of the PLA composites was slightly lower than PLA. Tomography scans revealed the fabrics were well oriented and some adherence between jute fabrics and PLA. Viscoelastic properties of PLA composites resulted in the reduction in storage modulus and the reduction in intensity in the damping factor attributed to segmental motions with no variations in the glass transition temperature. Flame retardant and spray adhesive on jute fabrics promoted better response to time of burning than PLA and PLA with modified fibers. The results presented in this work lead to the need for a more detailed investigation of the effect of plant fiber fabrics as reinforcement of 3D printed objects for industrial applications.

## 1. Introduction

Additive manufacturing (AM) of polymers is an automated process for producing three-dimensional objects from computer-aided design (CAD) data, and it is mostly used for prototyping that cannot manufacture one-piece products. The relevance of this technology has been constantly evolving over the years, and it is standardized by the common standards ISO/ASTM 52900:2015.

FFF, also known as fused deposition modeling (FDM), is the 3D printing of polymers based on the extrusion process. The object is built by depositing melted thermoplastic layer-by-layer through a heated nozzle onto the platform or over previously printed layers until the designed element is completed. Conventional-fused filaments, such as polyamide, acrylonitrile–butadiene–styrene (ABS), polyether ether ketone (PEEK), and PLA, are commercially available for domestic users. PLA is an aliphatic polyester corn starch-based thermoplastic and has been extensively examined in the literature as the most popular biodegradable material used for AM [1,2,3,4,5,6,7,8,9,10].

It is possible to find extensive literature, including several reviews, related to developing new experimental thermoplastic filaments prepared by the extrusion process to enhance the mechanical properties of 3D printed objects [1,2,3,11,12,13,14]. Part of this extensive research is aimed to find alternatives to recycled and biodegradable filaments for the sustainability of 3D printing [3,14,15].

Santana et al. [16] developed an exciting work of unifying the technology of textile concrete and additive manufacturing to develop composites of geopolymer matrix reinforced with printed polyethylene terephthalate glycol, commonly known as PETG, mesh. The composites were subjected to the notched prism bending test. The homogeneous reinforcement (volumetric polymer content of 4.75%) and the graduated (volumetric polymer content of 3.34%) produced an increase in toughness of 47 and 52 times, respectively, in addition to conserving the maximum load supported and reducing the volumetric content of the reinforcing material without compromising the mechanical performance of the composites.

Other works are focused on polymer modification and blends [4,5,17]. However, the most attractive seems to be the reinforced fused filaments with metal particles [18,19,20], clay minerals [6,21,22,23], graphene [8,24,25,26], glass [27], or carbon chopped fibers [28,29], and, more recently, continuous fibers [9,30,31].

A particular interest for several industrial applications is the use of continuous natural fibers as a substitute for glass fibers because of their mechanical and acoustic properties in combination with their end-of-life management and positive alternative to reduce carbon footprint. Ecological 3D printed objects are currently produced by combining long natural fibers and melted plastic, which are embedded in the hot block and deposed in a simultaneous manner [12,13,32,33,34]. Cellulosic fibers are widely available in most countries and are cost-effective with low density. They are biodegradable, renewable, non-hazardous, and non-abrasive. Furthermore, its specific mechanical properties are comparable to glass fibers. The purpose of adding these monofilament cellulosic fibers is to improve the mechanical properties of composite materials, including the construction industry, to improve the ductility and post-crack toughness of the composites [35]. Besides many advantageous properties of the natural fiber reinforced ecological composites, there are also some drawbacks, such as incompatibility with hydrophobic matrices, high water absorption, lower processing window, and bad surface appearance.

Numerous researchers have investigated PLA/natural fiber composites. Recently, Wis et al. developed over-molded jute / PLA fabric composites (OMC) on a laboratory scale. In that work, the authors developed hybrid organic composites prepared with thermoplastic composite technology and obtained lightweight composite components for structural parts. In this process, a reinforcing sheet composed of continuous glass or thermoplastic carbon fiber, called an organo-sheet, is over-molded using a thermoplastic polymer in an injection molding process. The composite sheets obtained are rigid, high-strength, and, at the same time, still have a detailed shape. The results obtained showed that the flexural modulus and the strength of OMC improved compared to pure PLA. Dynamic mechanical analysis showed that the thermomechanical resistance of PLA was improved for OMC [36]. Jerpdal et al. investigated the influence of overmolded temperature on tensile modulus, shrinkage, and strain for an insert made of self-reinforced polyethylene terephthalate (PET). The authors observed that a temperature above the glass transition temperature leads to relaxation of residual stresses and reduction in tensile modulus up to 18%. The study shows fascinating results, which may lead to new areas of application for self-reinforced PET [37].

Despite the extensive research and development effort of plant fiber-embedded polymers for 3D printing applications, there are no publications that deal with the use of fused filament deposition onto plant fiber fabrics; this is the research gap this work pretends to fill.

Among all-natural fibers, jute fiber seems to be a promising fiber with relevant research fields due to its good mechanical properties compared to other natural fibers, such as kenaf, sisal, and hemp [38,39]. The bag industry is the largest consumer of jute fibers because they represent an ecological option. However, many jute bags at the end of their lives are wasted and gone to the landfill every year.

This work aims to prepare new green composites through 3D printing PLA onto jute fabrics to evaluate the mechanical performance that allows discovering multiple industrial applications.

## 2. Experimental Section

### 2.1. Materials

**Figure 1:**
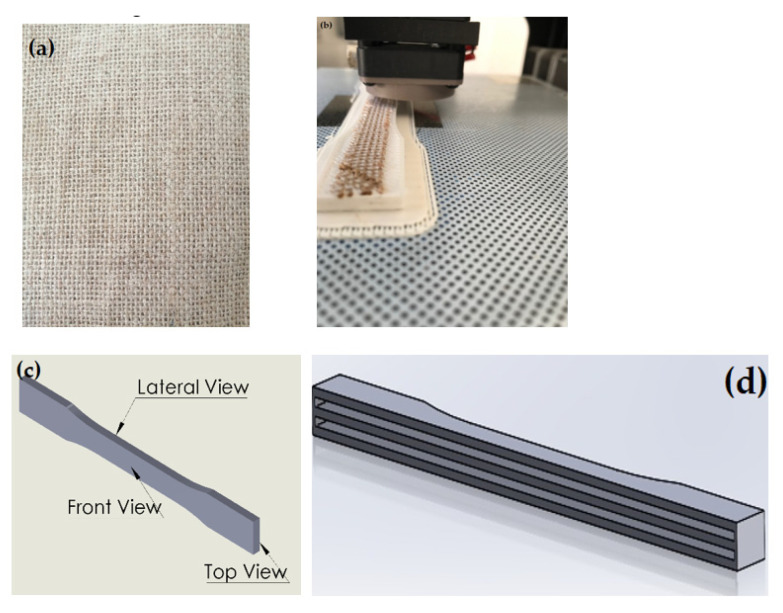
Photographs and schematic representations of: (**a**) jute fabrics, (**b**) 3D printing on jute fabrics, (**c**) tensile specimens’ solid-like configuration, and (**d**) specimens with gaps to place the jute fabrics. The solid-like picture also presents the viewing directions for tomography scans.

The commercial PLA-based filament with a diameter of 1.75 mm and a nominal density of 1.27 g/cm^3^ from 3D MARKET^®^ (Querétaro, Mexico) was used in this work. According to the supplier, this PLA filament has a tensile strength break between 55–65 MPa and a modulus of elasticity of 0.42 GPa. Natural jute fabrics with plain weave configuration and thickness of 0.91 mm (Figure 1) were obtained from bolsas publicitarias^®^ (Yucatán, Mexico) The fiber contains approximately 70 threads count, elastic modulus of 11 GPa, and tensile strength of 44 MPa. The mechanical properties were previously calculated following the methodology of the ASTM C1557.

### 2.2. 3D Printing Fabrication

A Zortrax M200 desktop 3D printer(Zortrax, Olsztyn, Poland) was used to print ASTM D638 Type I tensile specimens, previously modeled using SolidWorks software and exported to the 3D printing software as an STL file. Two different tensile specimen configurations were modeled. The first one was a solid-like specimen printed just with PLA. The second specimen included two longitudinal gaps of 0.91 mm, corresponding to the space to place the jute fabric, as schematized in Figure 1.

PLA was fused through a 0.4-mm-diameter nozzle at 200 °C and a printing speed of 50 mm/s over a bed platform heated at 50 °C. The specimens were built with 0.14-mm-layer thickness in a flat orientation with rectilinear pattern and an infill density of 90%. Jute fabrics dog bone geometry was cut using regular scissors and placed in the 0/90 direction (parallel to the uniaxial tension). The solid-like specimen was continuously printed, whereas the composite specimens required interrupting the 3D printing process to place the jute fiber fabric, as presented in Figure 1b. All composite specimens contained two jute fabric layers.

In this work, various strategies were used to evaluate the feasibility of increasing the mechanical properties of 3D printed composites. In this way, the characterization of these materials was carried out using the materials listed in Table 1.

Firstly, jute fabrics (J-M) were washed in an ionized water bath at 75 °C for 2 h, and dried at 85 °C for 2 h in an air convection oven. Afterward, jute fabrics were chemically treated with 5% NaOH and diazonium salt at alkali, acidic, and neutral media to increase compatibility with PLA.

Jute fabrics (J-R) were treated using a commercial flame retardant Flamebar S3 from Bollom fire protection. According to the supplier, the jute fibers were immersed for 12 h in a stainless steel container, having at least 70% of the solution, calculated on the submerged jute fabrics’ weight. Afterward, the jute fabrics were dried at 85 °C for 24 h in an air convection oven.

Jute fabrics (J-A) were sprayed with Hi-Tack 71 from 3M™, which is a mist aerosol adhesive recommended by 3M for its use for the manufacturing composites, including infusion and dry lamination. Spraying was carried out at a 45° angle before fiber placement during 3D printing.

The jute fabrics (J-MR) were firstly modified and subsequently treated with flame retardant. For the case of the J-MA, the J-M fabrics were treated and stored; then, the adhesive was applied to the fabric just a few minutes before to place it on the PLA during the 3D printing process.

### 2.3. Methods

Uniaxial tensile tests were performed according to ASTM D638-14 using a universal testing machine Instron^®^ 647(Instron^®^, Norwood, MA, USA) with a load cell of 10 kN. Ten specimens of each material were tested at room temperature (23 °C ± 2 °C) and at a 5-mm/min crosshead speed, and the curves showed in the results and discussion section are the representative curves based on the average behaviour revelaed during the tensile tests. Young’s modulus (E) and yield strength (σ_y_) were obtained from the engineering stress versus strain curves, and the elastic deformation was measured using a video extensometer MTS Advantage video extensometer (AVX) with 25 mm lens. The video-extensometer recognizes patterns on surfaces to acquire measurement data for strain calculations processed by MTS TestSuite™. Photographs of the failure zone after tensile tests were taken using a Zeiss stereomicroscope Discovery V8.

Three-dimensional computed tomography (CT) scans were performed in a GE phoenix v|tome|x instrument to visualize the inner of the 3D printed tensile specimens and detect adherence between PLA and jute fabrics. The analysis was conducted using X-Ray at 160 kV and 240 µA.

The viscoelastic behavior was evaluated in a Dynamical Mechanical Analysis DMA Discovery 850 from TA Instruments (Waters Corporation, Cary, NC, USA). The tests were performed in a single cantilever configuration at a frequency of 1 Hz and an amplitude of 30 µm. The specimens with 50 × 12 × 4 mm nominal dimensions were tested from 20 °C to 145 °C using a heating ramp of 5 °C/min. The curves of storage modulus (E’) and damping factor (tan δ) were obtained following the ASTM-D7028.

Flammability is highly interesting to analyze in ecological composite systems since it has become a crucial parameter in several industrial applications such as aeronautics, automotive, construction, or textile clothing. The flame retardant behavior of PLA and PLA composites was evaluated according to the methodology of chapter 3 of the Aircraft Materials Fire Test Handbook based on the FAR 25 Appendix F part III. The methodology proposed in this regulation can be used to predict the behavior of plastic or textile materials for diverse industrial sectors and aerospace applications. The test allows determining the burning speed of the specimens supported horizontally in a stainless steel cabin with air inlets on the top. Then, the flame (using methane gas) burns the specimen for 15 s. Subsequently, the ignition source is removed, and the test specimen is observed for time and extent of burning. An average burning rate is reported. Flammability tests were conducted in a multipurpose flammability chamber Deatak model MP-1 using high-purity methane gas (99%). Distance and time measurements were made with calibrated equipment, including Mitutoyo rulers with a resolution of 0.5 mm and a chronometer Control Company model 1025MX with a resolution of 0.1 s. The specimens with 50 mm × 13 mm × 4 mm nominal dimensions were conditioned at 21 °C and 55% humidity for at least 24 h before testing.

## 3. Results and Discussion

**Figure 2:**
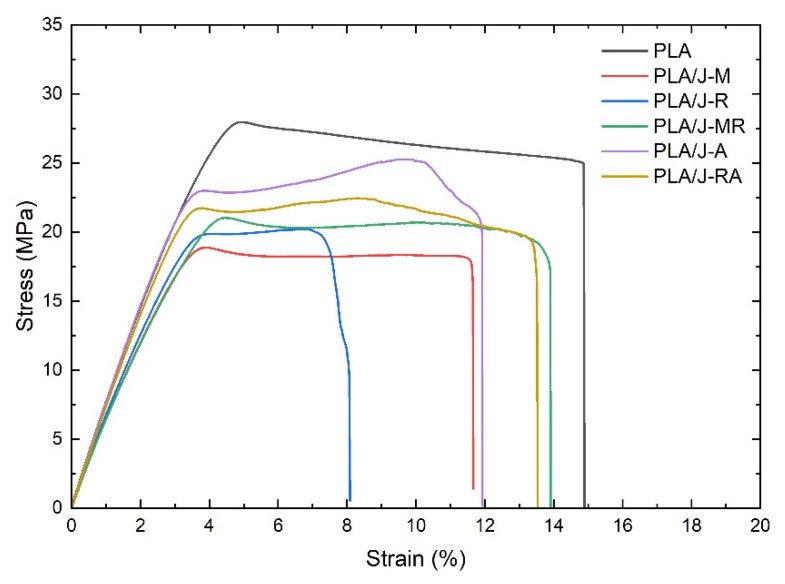
Stress vs strain curves corresponding to PLA and PLA composites.

The representative tensile engineering stress versus strain curves for PLA and PLA composites are shown in Figure 2.

It is possible to appreciate similar behavior for all materials evaluated in this work. The curves show a linear elastic region followed by diffusive necking and relatively low deformation until failure. It is worth noticing the presence of a shoulder in the plastic region developed for the composites. The adherence between jute fibers and PLA matrix requires a higher level of stress before failure, and it is the cause of the shoulder presence. The shoulder was evident in the PLA/J-A and PLA/J-RA composites.

The tensile parameters like E, σ_y_, and deformation at break (ε_b_) are summarized in Table 2.

The elastic modulus and strength of PLA are notably higher than PLA composites. The PLA/J-A and PLA/J-RA composites presented intermediate stiffness and strength values, and the rest had low mechanical properties.

On the other hand, PLA and PLA composites showed similar deformation values, except for the PLA/J-R, which showed low ductility and sudden failure. In general, the PLA specimens did not develop necking nor whitening. On the contrary, the specimens presented a homogeneous deformation with a brittle-like break during the tensile test.

It results show that the jute fiber is not compatible with PLA, which is confirmed by the low mechanical performance observed by the composite containing jute fiber with flame retardant (PLA/J-R). On the other hand, the modified treatment applied to the jute fabrics does not seem to influence the fiber-matrix interaction. Nonetheless, the combination of modified fibers followed by the flame retardant application seems to influence the molecular compatibility, which favors the mechanical properties of the PLA/J-MR composite. According to the mechanical results obtained, spray adhesive could prove to be the best strategy to achieve a better interaction between jute fabrics and PLA.

Ductility decreases because the fabrics restrict plastic deformation, although an increase in stiffness and strength is usually expected because the fibers promote the reinforcing effect of the polymeric matrix. Lack of compatibility, lack of adhesion, and distortion of fabrics also affect the mechanical performance of fiber-reinforced polymer systems.

**Figure 3:**
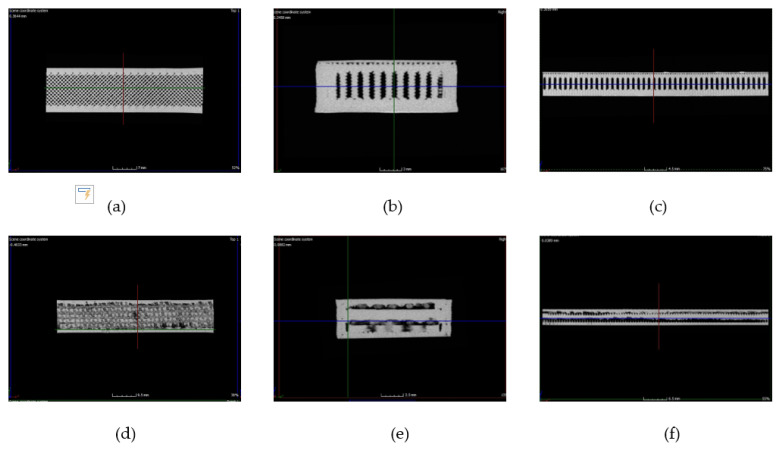
Tomography scans corresponding to PLA (upper) and PLA composite (bottom): (**a**) front view, (**b**) top view, (**c**) lateral view, (**d**) front view, (**e**) top view, (**f**) lateral view.

CT scan tomography is a powerful non-destructive testing tool for observing the disposition of the natural fiber fabrics into the 3D printing PLA specimens (Figure 3). We used CT scans to detect fabrics’ inner disruptions and reveal possible adhesion between fibers and PLA in this work.

Because of densities, PLA looks white, jute fiber appears grey, and the air is black. The inspection was performed in the gage zone of the tensile specimens before the test (Figure 1c).

The front view of PLA (Figure 3a) shows the rectilinear pattern with an infill density of 90%. This pattern develops the configuration of a stacking sequence similar to bridge pillars-like, as appreciated in the top and lateral views of the PLA specimens (Figure 3b,c, respectively). On the other hand, tomography of the PLA composite reveals that the jute fabrics are well-aligned, without distortions or fiber displacements, and are easily identifiable, as observed in the front view of Figure 3d. Furthermore, some interaction between fibers and PLA seems to occur, as observed in the top and lateral views (Figure 3e,f, respectively), although it is necessary to underline that the rectilinear pattern is altered or distorted when the fused filament is printed onto the fabrics. The previous promotes the shell-like appearance, compared to the top and lateral views of PLA and PLA composites in Figure 3. Considering that the jute fabric configuration is a conventional woven (not spread-tow), the fused filament is placed over the weft and warp surface with a different texture than PLA filament, which could be altering the 3D printing pattern and favoring some adherence between fibers and PLA that restrict the ductility during the tensile tests. 

**Figure 4:**
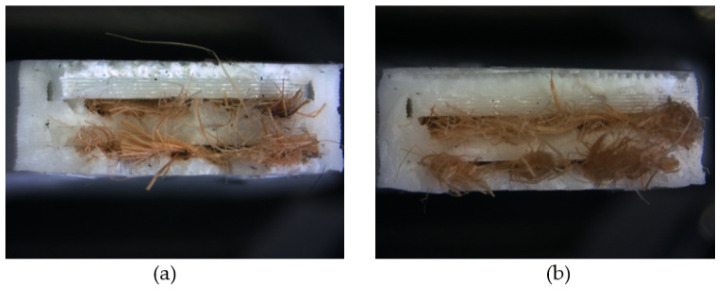
Photographs of the failure surface corresponding to: (**a**) PLA/J-M, (**b**) PLA/J-RA. All specimens failed similarly.

After the tensile test, the specimens broke suddenly due to the failure of the PLA matrix, keeping part of the jute fiber fabric together until its complete breakage, as can be seen in Figure 4.

**Figure 5:**
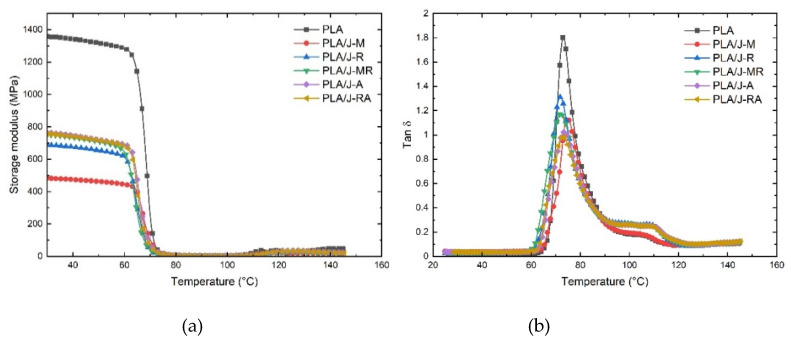
DMA curves corresponding to PLA and PLA composite: (**a**) storage modulus, (**b**) tang δ.

DMA provides relevant information on the viscoelastic behavior of polymers and composites. Storage modulus measures stress stored in the specimens as mechanical energy, while the damping factor is a typical measure of energy dissipation. DMA curves corresponding to storage modulus and damping factor are presented in Figure 5.

The storage modulus curve contains the glassy region, the leathery zone, and the rubbery plateau. In this work, the shape of the DMA curves was similar between PLA and PLA composites (Figure 5a). All materials show that the storage modulus remains practically constant until the onset temperature (T_onset_) indicates the leathery region’s starting point, where the modulus drops abruptly, followed by the rubbery plateau. Then, the storage modulus increases above 115 °C, which is associated with the cold crystallization of the amorphous part of PLA. The T_onset_ of PLA and some PLA composites is similar (between 60 °C and 62 °C). The PLA/J-M shows the higher Tonset (64 °C).

From 20 °C to 60 °C, the PLA presents a storage modulus of 1358 MPa, which is notoriously higher than the value obtained for the PLA composites (~757 MPa) at the same temperature range. The previous should imply that the jute fabrics do not act as reinforcement in the glassy region.

The damping factor or Tan δ curves of PLA and PLA composite are similar (72.8 to 73.2 °C). The height of the tan δ peak is associated with the chain mobility of the amorphous region in the polymer composites. In this study, the peak position at approximately 73 °C indicates that the glass transition temperature of PLA is not altered by the addition of jute fabrics but affected the chain mobility in the amorphous region due to the confinement effect resulting in the reduction in tan δ peak height.

According to the methodology of chapter 3 of the Aircraft Materials Fire Test Handbook, the material meets the approval criteria if three specimens of the same material show a burning rate of less than 63.5 mm/min, considering that the thickness of specimens must be less than or equal to 13.0 mm. In this work, the specimens were out of specifications. Nonetheless, the burn rates were obtained using a calibrated distance of 38.1 mm. A horizontal burning test was carried out for the burning time and burning rate of the PLA and PLA composites for the flammability properties at room temperature. Table 3 presents the results obtained.

**Figure 6:**
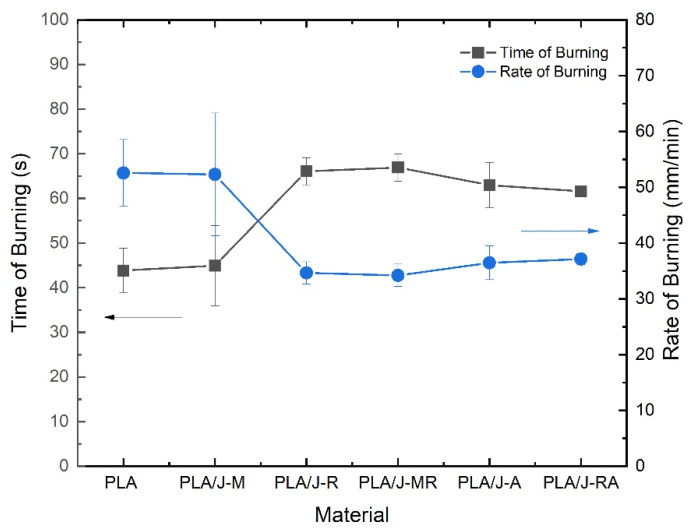
Time and rate of burning obtained for PLA and PLA composites.

It can be seen that the rate of burning of PLA and PLA composites meet the acceptance criteria of the reference standard, except for one value obtained for the PLA/J-M-1). Considering that this value is out of specifications and atypical data, it could be discarded. Figure 6 shows the time and rate of burning obtained for PLA and PLA composites.

It is possible to appreciate that the PLA and PLA composites containing modified jute fibers present low burning times (PLA/J-M). It means that the retardant treatment employed in this work induces a practical function on the flammability of PLA because the increase in burning time implies improvements in flame retardancy. Similarly, it appears that the spray adhesive exhibits some flame retardant effect since the time of burning exhibited for the PLA/J-A and PLA/J-RA displayed similar results to the PLA/J-R.

## 4. Conclusions

The results obtained in this work confirm the feasibility of producing 3D printing objects using plant fiber fabrics as filler. PLA-fused filament was successfully deposed onto natural fiber fabrics to print dog bone tensile specimens for characterization. In the first instance, the mechanical properties obtained for the PLA composites are not superior to PLA. However, viscoelastic properties allowed to identify the jute fabrics hinder the molecular motions, and the glass transition temperature remains.

Flame retardant and spray adhesive on jute fabrics promoted better composites’ responses than PLA and PLA with modified fibers. One characteristic feature observed is the flame retardancy which increases because of the combined effect between jute fabrics and PLA, forming dense char which further resists the flame propagation.

The results obtained allow us to visualize the potential use of flame retardants for this composite material and the vast flammability analysis that can be deepened through cone calorimeter or limited oxygen index tests.

The use of plant fiber fabrics as reinforcement of 3D printed objects is a vast field of research. This work confirms the feasibility that plant fiber fabrics can be used as effective reinforcement. Other advantages of re-using this ecological waste material are that natural fibers are much less dense than synthetic fibers and polymers, which leads to developing lightweight composites and less raw material consumption for 3D printing parts and components. The results presented in this work lead to the need for a more detailed investigation of the effect of plant fiber fabrics as reinforcement of 3D printed objects for industrial applications. There are many questions about these new ecological materials, such has how the addition of distinct flame retardants, the fabric configuration, or different treated fibers can affect their mechanical performance. Some of these questions are being studied by our research group.

## Figures and Tables

**Table 1:** Materials description and their reference used in this work.

Reference	Description
PLA	PLA
PLA/J-M	PLA/jute fiber modified
PLA/J-R	PLA/jute fiber with flame retardant
PLA/J-MR	PLA/jute fiber modified and flame retardant
PLA/J-A	PLA/jute fiber with adhesive
PLA/J-RA	PLA/jute fiber with flame retardant and adhesive

**Table 2:** Mechanical parameters of PLA and PLA composites.

Material	E (GPa)	σ_y_ (MPa)	σ_b_ (MPa)	ε_b_ (%)
PLA	1.98 ± 0.02	27.93 ± 1.25	25.13 ± 1.16	14.76 ± 0.98
PLA/J-M	1.22 ± 0.23	18.81 ± 3.76	18.24 ± 1.98	11.48 ± 1.25
PLA/J-R	1.41 ± 0.13	19.84 ± 1.86	19.72 ± 1.98	7.51 ± 2.68
PLA/J-MR	1.26 ± 0.46	21.11 ± 2.33	19.41 ± 1.98	13.68 ± 1.26
PLA/J-A	1.83 ± 0.19	22.97 ± 2.16	21.88 ± 1.98	11.76 ± 1.89
PLA/J-RA	1.62 ± 0.16	21.78 ± 1.89	19.76 ± 1.98	13.36 ± 1.36

**Table 3:** Flammability test data report for PLA and PLA composites.

Material	Length (mm)	Width (mm)	Thickness (mm)	Burning Distance (mm)	Time of Burning (s)	Rate of Burning (mm/min)
PLA-1	51.3	13.23	4.77	38.1	49.55	46.14
PLA-2	50.49	13.27	4.75	38.1	41.65	54.89
PLA-3	50.66	13.24	4.74	38.1	40.33	56.68
PLA/J-M-1	50.36	13.69	4.94	38.1	35.08	65.17
PLA/J-M-2	51.16	13.76	5.07	38.1	48.88	46.77
PLA/J-M-3	51.59	14.03	5.13	38.1	50.83	44.97
PLA/J-R-1	51.75	13.67	4.84	38.1	64.57	35.40
PLA/J-R-2	50.72	13.53	4.81	38.1	63.62	35.93
PLA/J-R-3	49.95	13.54	5.16	38.1	70.03	32.64
PLA/J-MR-1	51.52	13.73	5.24	38.1	67.5	33.87
PLA/J-MR-2	51.7	13.41	5.11	38.1	63.29	36.12
PLA/J-MR-3	50.64	13.71	5.24	38.1	69.99	32.66
PLA/J-A-1	50.21	13.38	4.99	38.1	67.46	33.89
PLA/J-A-2	50.09	13.65	5.08	38.1	57.54	39.73
PLA/J-A-3	50.07	13.5	4.98	38.1	63.95	35.75
PLA/J-RA-1	50.38	13.45	5.01	38.1	62.5	36.58
PLA/J-RA-2	48.16	13.39	4.97	38.1	62.21	36.75
PLA/J-RA-3	50.16	13.4	5.28	38.1	60.11	38.03

## Data Availability

This study did not report any data.
